# Perspectives on the α5 nicotinic acetylcholine receptor in lung cancer progression

**DOI:** 10.3389/fcell.2025.1489958

**Published:** 2025-03-12

**Authors:** Jiaying Cai, Jingting Wang, Zengping Wang, Jing Wang, Yanfei Jia, Xiaoli Ma

**Affiliations:** Research Center of Basic Medicine, Central Hospital Affiliated to Shandong First Medical University, Jinan, Shandong, China

**Keywords:** α5-nAChR, growth, epithelial-mesenchymal transition (EMT), immune escape, lung cancer

## Abstract

Nicotinic acetylcholine receptors (nAChRs) are widely expressed in a variety of cell types and are involved in multiple physiological regulatory mechanisms in cells, tissues and systems. Increasing evidence suggests that the α5 nicotinic acetylcholine receptor (α5-nAChR), encoded by the CHRNA5 gene, is one of a key mediator involved in lung cancer development and immune responses. Several studies have shown that it is a regulator that stimulates processes via various signaling pathways, including STAT3 in lung cancer. In addition, α5-nAChR has a profound effect on lung immune response through multiple immune-related factor pathways. In this review, we focus on the perspectives on α5-nAChR in lung cancer progression, which indicates that targeting α5-nAChR could provide novel anticancer and immune therapy strategies for lung cancer.

## 1 Introduction

Lung cancer has a high incidence rate and mortality among all kinds of malignant tumors (Thai et al., 2021). Numerous epidemiological researches conducted worldwide have confirmed that smoking is the biggest risk factor for lung cancer (Balata et al., 2019; Gallaway et al., 2019), and there are more than 50 kinds of substances in tobacco that can cause cancer, while nicotine is the main addictive factor of smoking, which can promote the progression of lung tumors. Researches indicate that nicotine possesses multifaceted impacts, significantly stimulating cancer cell growth, motility, and immune modulation while occupying a central position in facilitating tumor dissemination, invasive behavior, and the epithelial-mesenchymal transition (EMT) process (Zhang et al., 2016).

The multiple functions of nicotine are shaped by its combination with various nicotine acetylcholine receptors (nAChRs). nAChRs are pentameric structures consisting of five transmembrane units that form Na^+^, K^+^ and Ca^2+^ permeable cation channelsand are expressed in the nervous system and many non-neuronal tissues) (from monovalent Na^+^ and K^+^ ions to divalent Ca^2+^ ions). Ca^2+^ ions, in turn, affect signal transduction (Hogg et al., 2003). The ionic characterisation of nAChRs refers to the fact that binding of ligands to nAChRs activates a metastatic shift in the receptor, leading to channel opening and altered ion fluxes in the cell. nAChRs consist of α1∼α10 subunits, four β1∼β4 subunits, δ, ε and γ subunits, arranged in the form of heteromeric or homomeric structures, thereby underpinning their multifaceted roles (Picciotto and Kenny, 2021; Papke, 2014). Homomeric nAChRs consist of five identical α subunits particularly (α7 and α9) (Zoli et al., 2018). Heteromeric nAChRs encompass a blend of α and β subunits (Niu and Lu, 2014). These various subunits of nAChRs exhibit differential expression patterns across diverse tissues and organs. It has also been shown that ligand binding is also involved in metabolic channel reactions (Sinclair and Kabbani, 2023). Genome-wide association studies (GWAS) reported that a strong correlation between α5-nAChR and both lung cancer predisposition and nicotine addiction (Falvella et al., 2010; Hung et al., 2008; Berrettini et al., 2008; Liu et al., 2010). α5-nAChR, encoded by CHRNΑ5 gene, holds pivotal significance in modulating tumor growth, apoptosis and angiogenesis (Zhou et al., 2020). Additionally, it stands as an established indicator of smoking-related risks (Sun and Ma, 2015; Jensen et al., 2015; Lassi et al., 2016). Our studies showed that nicotine activates α5-nAChR triggering downstream signaling cascades in lung cancer cells. This activation process facilitates a cascade of effects, including the proliferation, invasion, and immune escape of lung cancer (Zhu et al., 2022a; Zhang et al., 2017; Sun et al., 2017; Jia et al., 2022; Zhang et al., 2021a).

## 2 α5-nAChR partaked lung cancer growth

α5-nAChR plays a pivotal role in the development of lung cancer (Bele et al., 2024; Improgo et al., 2010a). Investigations revealed a robust association between α5-nAChR and STAT3 among the relevant factors affecting lung adenocarcinoma (LUAD) progression (Zhang et al., 2017). STAT3, a pivotal transcription factor, coordinates multiple cellular processes, notably cell growth and apoptosis, and governs an array of genes crucial for cancer cell survival, proliferation, invasion, metastasis, drug resistance and immune evasion (Song et al., 2011). This factor is responsive to cytokines, growth factors, and exogenous carcinogens such as nicotine (Zhang et al., 2017; Yu et al., 2009; Xu et al., 2019). In LUAD cells, nicotine stimulates α5-nAChR, initiating a cascade where STAT3 signaling, which induces STAT3 to bind to gene promoters and modulate gene transcription, thereby promoting cell proliferation and associated processes. Notably, JAK2 activation modulates STAT3 phosphorylation, it has been shown that nicotine interacts with cell surface α5-nAChR to activate various signaling pathways, including the JAK2/STAT3 pathway, thereby affecting lung cancer progression (Zhang et al., 2017).

The phosphoinositide 3-kinase (PI3K)/protein kinase B (PKB, also known as AKT) signaling pathway plays a crucial role in the development of lung cancer by promoting cell survival, tumorigenesis and treatment resistance (Yu and Cui, 2016; Hoxhaj and Manning, 2020; Yang et al., 2019). Nicotine has been shown to affect non-small cell lung cancer (NSCLC) by activating AKT pathway via α5-nAChR (Ma et al., 2014). α5-nAChR participated in nicotine-induced cell proliferation through PI3K/Akt axis, thereby inducing HIF-1α and VEGF expression. The HIF-1α pathway holds a central position in the process of carcinogenesis (Wang et al., 2013). HIF-1α, serving as a pivotal transcription factor, engages in heterodimerization with the constitutively expressed HIF-1β subunit. This complex subsequently stimulates the transcription of numerous genes, among which VEGF stands out as a key player in promoting tumor cell proliferation. Nicotine engages with the α5-nAChR on the surface of lung cancer cells, activating the Akt signaling pathways, upregulating HIF-1α signaling, and increasing VEGF, thereby accelerating the growth of lung cancer (Carlisle et al., 2007).

Our comprehensive gene expression profile shows the pivotal function of α5-nAChR in cell cycle progression, DNA replication, and apoptosis by regulating cell cycle-associated gene expressions (cyclin D1, E2, and D3) (Sun et al., 2017). The D-type cyclin proteins, including cyclinD1, cyclinD2, and cyclinD3, promote cell division through activation of CDK4 as well as CDK6, which in turn phosphorylates the RB family (Malumbres and Barbacid, 2009). Initiated during the G1 phase, cyclin D synthesis propels the critical G1/S phase transition. D-type cyclin proteins and cyclin-dependent kinases (CDKs) are central molecules in the overall cell cycle regulation mechanism, driving cell proliferation. α5-nAChR exerts a suppressive effect on the expression of cyclin D1, E2 and D3, thereby promoting the transition of lung cancer cells from G_0_/G_1_ phase to S phase, hinting at its potential modulation of cell cycle checkpoints, and its role in tumor progression should not be ignored. Furthermore, nicotine induces proliferation of human breast cancer cells through downregulation of nicotinic receptors and cyclin D3 (Chen et al., 2010). Survivin, a tumor-specific member of the apoptosis inhibitor protein family, which is expressed only in tumor and embryonic tissues, intimately ties tumor differentiation, proliferation, invasion and metastasis. A positive correlation exists between α5-nAChR and survivin expression levels. The co-expression of α5-nAChR and survivin can significantly promote the proliferation and survival of lung cancer cells, significantly reduce the survival rate of patients, and jointly contribute to unfavorable prognoses (Zhang et al., 2021a). Clinical analysis indicated that high level of tumor α5-nAChR is correlated with poor survival rates of LUAD patients, particularly in those expressing wild-type EGFR (Wang et al., 2020). The study identified α5-nAChR as an essential mediator for low-dose nicotine-dependent LUAD progression possibly through signaling crosstalk with EGFR, supporting the involvement of environmental smoke in tumor progression in LUAD patients. Spinosad disrupted the interaction between α5-nAChR and EGFR, thereby inhibiting the formation of downstream complexes and activation of the EGFR signaling pathway (Zou et al., 2024). These studies offer theoretical and experimental foundation for novel LUAD treatments.

## 3 α5-nAChR involved in lung cancer invasion and EMT

Lung cancer, as a highly aggressive malignant tumor and its progression is fraught with danger that is often invasive and metastatic at the time of diagnosis. In order to gain the ability to move and invade, cancer cells must discard many of their epithelial phenotypes, change their phenotype, detach from the epithelial layer, and undergo a series of significant alterations, a process called epithelial-mesenchymal transition (EMT) (Lamouille et al., 2014). EMT, a pivotal biological process, enables epithelial-derived tumor cells to attain migratory and invasive capabilities, and it is one of the hotspots in the study of tumor metastasis, as well as holding significance in embryonic development, tissue reconstruction, and chronic inflammation processes (Dongre and Weinberg, 2019; Pastushenko and Blanpain, 2019; Taki et al., 2021). Acetylcholine and α5-nAChR interaction diminished the expression of E-cadherin, a marker of EMT, along with an increase of N-cadherin, vimentin, β-catenin, and ZEB-1 expression (Fu et al., 2024). It was shown that α5-nAChR regulates the expression of STAT3 and Jab1/Csn5, significantly modulates the expression of EMT markers, and affects lung cancer invasion and metastasis (Chen et al., 2020).

EMT represents the replicative biological process by which cancer cells acquire invasive and motile capabilities, and this includes a number of important effector molecules, the most important of which is matrix metalloprotein (MMP), which is a key factor in the extracellular matrix degradation (Almutairi et al., 2023). The interaction of acetylcholine with α5-nAChR triggers the activation of the STAT3/DNMT1 signaling axis. DNMT1 is able to mediate the CpG islands methylation, thereby controlling the promoter methylation of the tumor suppressor gene FHIT in cancer cells, thereby inducing an increase in the expression of MMP-9 and vimentin, and promoting the invasive metastasis of lung cancer (Jiao et al., 2023).

The activity of Jab1 involved in diverse tumorigenic pathways, positioning it as a promising therapeutic target in smoking-related lung cancer (Liu et al., 2018). Notably, the expression levels of Jab1 display a positive association with α5-nAChR. α5-nAChR via STAT3/Jab1 signaling cascade facilitates EMT and metastasis in lung cancer cells, accompanied by enhanced N-cadherin and vimentin expression (Chen et al., 2020). Our investigation introduces a novel perspective, the involvement of the α5-nAChR/Jab1 signaling axis in lung cancer EMT and metastasis may provide a new tumor-targeting strategy for lung cancer treatment.

α5-nAChR mediates PLEK2 expression, a member of the pleckstrin protein family discovered in platelets and leukocytes, within the context of lung cancer through the regulation of STAT3. PLEK2 is a cytoskeletal protein that is mainly involved in the reorganization of cytoskeletal proteins, cellular stretch, and the regulation of migration, all of which are intricately linked to EMT. Nicotine interacts with α5-nAChR on the surface of lung cancer cells, activating the α5-nAChR/PLEK2 signaling pathway, which is crucial for cellular migration, invasion and differentiation (Li et al., 2024).

The MAPK/ERK signaling pathway has attracted much attention in the development of oncology therapies, and it plays an important role in cell growth, and invasion (Ullah et al., 2022). The MAPK signaling pathway is present in most cells and plays a critical role in transducing signals from extracellular stimuli into the cell and its nucleus, thereby triggering cellular biological responses. The MAPK/ERK signaling pathway can be found in many human tumor tissues in a state of abnormally high expression and activity, promoting tumor cell proliferation, differentiation and invasion (Ma et al., 2023; Cui et al., 2023; Fang and Richardson, 2005). α5-nAChR participates in the signaling pathway, which affects lung cancer invasion (Ma et al., 2014). α5-nAChR promotes tumor cell proliferation by promoting ERK phosphorylation and upregulating HIF-1α, which in turn affects VEGF, the most characteristic regulator of hypoxia.

## 4 α5-nAChR mediated lung cancer immune escape

With the rapid development of tumor immunotherapy, lung cancer treatment has stepped into a new era of immunotherapy, which affects all aspects of tumourigenesis and treatment response (Zhu et al., 2022a; Kang et al., 2023). At the nexus of numerous oncogenic signaling cascades, STAT3 assumes a pivotal role in modulating the anti-tumor immune response (Zou et al., 2020). Extensively activated within both neoplastic and non-neoplastic cells of the tumor microenvironment, STAT3 critically suppresses the expression of vital immune activators while concurrently fostering the generation of immunosuppressive factors (Wang et al., 2018). STAT3 can be activated in a variety of human tumors, and STAT3 overexpression has been observed in a variety of patient-derived tumor tissue samples. Numerous studies strongly supports blocking STAT3 activation using inhibitors or knockout systems as an attractive therapeutic target for cancer and other human diseases (Song et al., 2011). The investigation revealed that the NLRP3 promoter contains a binding motif for STAT3, enabling STAT3 to specifically interact with this promoter region (Zhang et al., 2021b). The regulation of STAT3 by α5-nAChR serves as a mediator in modulating the expression levels of NLRP3, thereby affecting the progression of lung cancer (Jia et al., 2022). Among the diverse classes of inflammasome, NLRP3 stands out as the most extensively characterized entity (Dey Sarkar et al., 2021). The constitution of this complex arises through the integration of a nucleotide-binding and oligomerization domain (NOD)-like receptor NLRP3, alongside the adaptor protein apoptosis-associated speck-like protein containing a caspase recruitment domain (ASC), and the precursor form of caspase-1, pro-caspase-1 (Huang et al., 2021). This complex promotes the activation of caspase-1, a pivotal event that triggers the maturation and subsequent release of pro-inflammatory cytokines, namely, IL-1β and IL-18, while also instigating cellular pyroptosis (Holbrook et al., 2021). This suggests that NLRP3 is involved in α5-nAChR-mediated lung cancer progression and provides a new molecular mechanism for targeting the α5-nAChR/STAT3/NLRP3 axis against lung cancer.

Within the intricate tumor microenvironment, tumor-associated macrophages (TAMs) constitute the predominant immune cell population, intimately participating in tumor progression and metastasis dissemination (Condeelis and Pollard, 2006; Mantovani et al., 2008). CD47 is a checkpoint for phagocytosis in macrophages and a therapeutic target for several cancer types (Jiang et al., 2021). It interacts with macrophage signal regulatory protein α (SIRPα) to inhibit TAM phagocytosis of tumor cells and induce immune escape (Kang et al., 2023). α5-nAChR mediated immune escape via TAM and mediates CD47 expression through STAT3 signaling affecting lung cancer migration, invasion, and immune escape (Ma et al., 2014). Extensive research underscores ubiquitous overexpression of CD47 in a wide range of tumors, with its heightened expression levels prognosticating dismal survival outcomes for cancer patients (Matlung et al., 2017; Kang et al., 2024). In α5-nAChR-mediated immune escape of CD47, TAM decreases cytokines secreted by M1-type macrophages with pro-inflammatory and immunosurveillance functions, and increases cytokines secreted by M2-type macrophages with anti-inflammatory, pro-tumor growth and immunosuppressive functions.

The CD274 gene encodes programmed death ligand 1 (PD-L1), which not only exerts a suppressive influence on the immune system but also holds a unique tumor-intrinsic function in fostering tumor growth, facilitating metastasis and conferring resistance to therapeutic interventions. PD-L1 is seen as a crucial mediator in the transmission of intrinsic signals to accelerate tumor progression (Nguyen et al., 2019; Yi et al., 2021). Notably, the expression of α5-nAChR displays a positively correlated with PD-L1 levels. α5-nAChR mediates the expression of PD-L1 through STAT3, and subsequently PD-L1 binds to PD-1, which mediates the activity of Tergs, CTLs, and NK cells, which influences the progression of lung cancer and participates in immune escape (Zhu et al., 2022b). Jab1 is also involved in α5-nAChR mediated PD-L1 lung carcinogenesis. Recent investigations have demonstrated Jab1 to be plausible in smoking-induced lung carcinogenesis, serving as a pivotal modulator engaged in multifarious tumorigenic pathways (Liu et al., 2018; El-Aarag et al., 2017). Its significance extends to crucial functions in cancer initiation, advancement, and clinical outcomes. In addition, Jab1 is indispensable for maintaining PD-L1 stability in cancer cells, where it inhibits the ubiquitination and degradation of PD-L1, thereby suppressing tumor-infiltrating cytotoxic T-cell immune responses (Lim et al., 2016) and fostering tumor progression and migration (Liu et al., 2020; Ruan et al., 2021). These findings unravel novel crosstalk between α5-nAChR and PD-L1, underpinning lung cancer cell growth and progression, potentially presenting a novel therapeutic avenue for lung cancer diagnosis and immune-based therapies.

Ly6E, belonging to the Ly6 family, serves as an indicative biomarker for adverse prognosis in nicotine-induced lung carcinogenesis, intricately modulating the TGF-β1/Smad signaling cascade. Its involvement in human malignant neoplasms underscores its potential therapeutic target for cancer immunotherapy, as it fosters cancer progression, immune escape and therapeutic resistance through TGF-β signaling pathways (Alhossiny et al., 2016). Notably, nicotine elicits an upregulation of α5-nAChR, Ly6E, phosphorylated Smad3 (pSmad3), Zeb1, N-calmodulin, and vimentin in lung cancer cells (Zhang et al., 2022). This process involves nicotine activates to α5-nAChR on the cell surface, subsequent interaction with Ly6E, and activates TGF-β1/Smad signaling to promote lung cancer cell motility. The collaboration between Ly6E and α5-nAChR in lung cancer directs TGF-β1/Smad3 signaling, modulating neoplasms migration (Zhang et al., 2022). α5-nAChR mediates Ly6E, phosphorylation of the TGF-β1 downstream molecule Smad3, the epithelial-mesenchymal transition (EMT) marker Zeb1, N-calmodulin, and vimentin expression in NSCLC cells (Zhang et al., 2022). This discovery marks the pioneering evidence linking α5-nAChR and Ly6E expression in lung carcinogenesis, positioning these molecules as promising targets for the lung cancer-specific therapies. Furthermore, the interaction between α5-nAChR and immune-related molecules accentuates its role in tumor immune defense, offering insights into potential therapeutic avenues for nicotine-associated lung cancer (Tang et al., 2020). This multifaceted understanding underscores the significance of investigating these molecular interactions in the context of lung carcinogenesis and immunotherapy.

## 5 Discussion

Genome-wide association studies (GWAS) have shown that the chromosomal region of 15q25, which contains several nicotinic acetylcholine receptor-encoding genes that are associated with nicotine addiction (Thorgeirsson et al., 2008) and smoking behaviour (Liu et al., 2010), is associated with lung cancer risk. One of these susceptibility loci, rs16969968, a non-synonymous variant polymorphism located in the exon five of CHRNA5 15q25 resulting in a change in amino acid at residue 398th of CHRNA5 from Asp to Asn (Hung et al., 2008; Zhou et al., 2020), has been identified by GWAS as a risk locus for lung cancer (Weiss et al., 2008). In addition, the 15q25 region also includes the CHRNA3, CHRNB4 cluster encoding α3-nAChR and β4-nAChR. These receptors, activated by acetylcholine, nicotine, and their byproducts, play a pivotal role in nicotine dependence and the pathogenesis of tobacco-associated disorders. α5-nAChR may affect the functional properties of nAChRs in several ways, including: (i) altering the potency and efficacy of ligands; (ii) affecting the receptor’s Ca^2+^ permeability; (iii) altering the receptor’s desensitization properties; (iv) regulating receptor expression, posttranslational processing, and/or trafficking to the cell membrane; and (v) modulating Ca^2+^-independent downstream signaling. α5-nAChR modulates α4β2∗ and α3β4∗receptors at the cellular level (Improgo et al., 2010b; Scholze and Huck, 2020). By far, α5-nAChR’s role in cell signaling is not clearly identified. Although genetic polymorphism in the gene of this subunit or mRNA downregulation by RNA interference affects the function of nAChRs, it is not clear if this is a direct or an indirect effect.

The signaling pathways associated with α5-nAChR are pivotal in the progression of cancer (Sun et al., 2017). Beyond this subtype, other isoforms of the nAChRs contribute significantly to lung carcinogenesis. Studies have demonstrated the function of α7-nAChR and α9-nAChR in promoting the growth of non-small cell lung cancer cells, which further extend their influence to other malignancy types (Mucchietto et al., 2018). α7-nAChR enhances colorectal cancer cell migration via nicotine-nitrosated derivative of NNK (Wei et al., 2009); parasympathetic nerves might promote CRC (colorectal cancer) progression through α9-nAChR; α9-nAChR is overexpressed in smoking-associated breast cancer and plays an crucial role in nicotine-induced transformation of normal human mammary epithelial cells (Lee et al., 2010). In addition, α9-nAChR is highly expressed in triple-negative breast cancer (TNBC) and affects cancer metastasis (Liao et al., 2023). Multiple subtypes of nAChRs also have their own distinct roles in the immune response to a wide range of diseases (Pechlivanidou et al., 2023). α7-nAChR and α9-nAChR are ubiquitous in various immune cell types and are involved in immune function and in the effects of immunobiology (Shelukhina et al., 2023).

Furthermore, α5-nAChR involves in chronic stress-mediated lung adenocarcinoma (Jiao et al., 2023). Chronic stress has become a common characteristic among individuals in contemporary society. It elicits a cascade of cancer-promoting factors, modulating tumor initiation, progression, prognosis, and the intricacies of the tumor microenvironment (Tian et al., 2021). Accumulating evidences underscore the link between chronic stress and tumorigenesis (Eckerling et al., 2021). Chronic stress can activate the release of the neurotransmitter acetylcholine (ACh) by parasympathetic postganglionic nerve fibers, ligands for nAChRs, which subsequently triggers the α5-nAChR/FHIT signaling cascade (Jiao et al., 2023). Previous studies have shown that FHIT is significantly associated with chronic stress-induced depression (Direk et al., 2017). Recent studies have shown that nicotine contributed to tumor progression through epigenetics, microbial metabolism pathway (Ohue-Kitano et al., 2024; Guan et al., 2024; Dawes et al., 2021), while the role of α5-nAChR on epigenetic regulation, microbiome, and metabolic reprogramming in nicotine-associated lung cancer remain to be studied.

## 6 Conclusion

**FIGURE 1:**
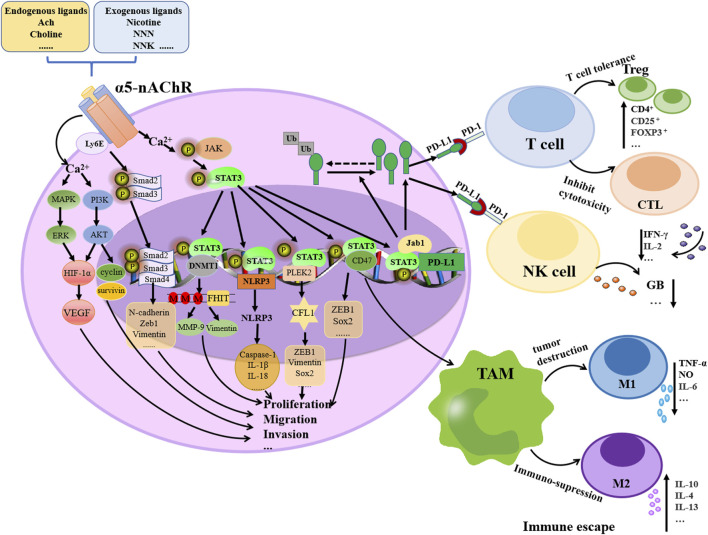
Schematic representation of the molecular mechanism of α5-nAChR contributed to lung cancer progression. Nicotine, other endogenous or exogenous ligands, or environmental factors by binding to α5-nAChR induce lung cancer proliferation, migration, invasion and immune escape by activating signaling cascades, e.g., MAPK/ERK, PI3K/AKT, Smad signaling, JAK/STAT3, Jab1/PD-L1 and STAT3/CD47 pathways.

α5-nAChR expression could be influenced by nicotine, endogenous acetylcholine, other ligands or environmental factors, potentially contributing to lung tumorigenesis, immune modulation via diverse signaling cascades (Figure 1). Accumulating evidence suggests that different characteristics of tumor cells, such as proliferation, metastasis, apoptosis and chemoresistance, are closely related to inflammatory responses. As changes in α5-nAChR lead to different alterations in multiple signaling factors downstream of it, which in turn affect the progression of lung cancer, this suggests that α5-nAChR may be a potential therapeutic target and prognostic biomarker for lung cancer (Table 1).

**TABLE 1:** α5-nAChR in lung cancer progression.

Function	α5-nAChR and signaling pathways	Changes	References
Proliferation	JAK2/STAT3	↑α5-nAChR:↑p-STAT3, p-JAK2	Zhang et al. (2017)
	PI3K/AKT	↑α5-nAChR:↑p-Akt, HIF-1α, VEGF	Ma et al. (2014)
	cyclin	↑α5-nAChR:↑cyclin D1, cyclin E2, cyclin D3; ↓G_0_/G_1_ phase to S phase	Sun et al. (2017)
	survivin	↑α5-nAChR:↑survivin; ↓apoptosis	Zhang et al. (2021a)
Invasion	DNMT1/FHIT	↑α5-nAChR:↑DNMT1, p-STAT3, vimentin, MMP-9; ↑migration; ↓FHIT	Jiao et al. (2023)
	Jab1/Csn5	↑α5-nAChR:↑p-STAT3, Jab1, EMT; ↑migration	Chen et al. (2020)
	STAT3/PLEK2	↑α5-nAChR:↑PLEK2, Zeb1, CFL1, CD44, Sox2; ↑migration, stemness ↓PLEK2, CFL1	Li et al. (2024)
	MAPK/ERK	↑α5-nAChR:↑p-ERK1/2, HIF-1α, VEGF	Ma et al. (2014)
Immune escape	STAT3/NLRP3	↑NLRP3:↑migration, invasion	Jia et al. (2022)
	STAT3/CD47	↑α5-nAChR: ↑CD47, IL-10, p-STAT3, ZEB1, Sox2; ↓TNF-α; ↑proliferation, migration, invasion, stemness	Kang et al. (2023)
	STAT3/PD-L1 OR STAT3/Jab1-PD-L1	↑α5-nAChR: ↑p-STAT3, Jab1, PD-L1, Tregs; ↓IFN-γ, GB	Zhu et al. (2022b)
	Ly6E	↑α5-nAChR: ↑Ly6E, p-Smad3, Zeb1, N-cadherin, vimentin; ↑migration, invasion, metastasis	Zhang et al. (2022)

Despite the progress in α5-nAChR research, several limitations require resolution. Evidences suggested that α5-nAChR promotes lung carcinogenesis through PI3K/AKT and MAPK signaling pathways, yet conflicting results from various experimental models and conditions challenge this results (Bele et al., 2024; Cheng et al., 2020). The tumor heterogeneity-driven spatiotemporal dynamics of α5-nAChR expression remain poorly characterized, necessitating advanced multi-omics integration (e.g., spatial transcriptomics and proteomics) for systematic elucidation. Furthermore, clinical translation lags behind preclinical findings, with insufficient clinical trials and validation studies. Therefore, the study of α5-nAChR requires: (1) Standardization of experimental models and detection methods to reduce outcome heterogeneity; (2) Validation of clinical relevance using organoids and patient-derived xenograft (PDX) models (3) Multi-omics integration (e.g., single-cell transcriptomics, epigenetics, and metabolomics); to dissect the spatiotemporal regulatory networks of α5-nAChR. The in-depth study of the molecular mechanism of α5-nAChR provides precise intervention strategies for specific patient subgroups.
